# Falls Risk in Relation to Activity Exposure in High-Risk Older Adults

**DOI:** 10.1093/gerona/glaa007

**Published:** 2020-01-16

**Authors:** Silvia Del Din, Brook Galna, Sue Lord, Alice Nieuwboer, Esther M J Bekkers, Elisa Pelosin, Laura Avanzino, Bastiaan R Bloem, Marcel G M Olde Rikkert, Freek Nieuwhof, Andrea Cereatti, Ugo Della Croce, Anat Mirelman, Jeffrey M Hausdorff, Lynn Rochester

**Affiliations:** 1 Translational and Clinical Research Institute, Faculty of Medical Sciences, Clinical Ageing Research Unit, Campus for Ageing and Vitality, Newcastle University, Newcastle upon Tyne, UK; 2 School of Biomedical Sciences, Newcastle University, Newcastle upon Tyne, UK; 3 School of Clinical Sciences, Auckland University of Technology, New Zealand; 4 KU Leuven, Department of Rehabilitation Sciences, Neuromotor Rehabilitation Research Group, Belgium; 5 Department of Neuroscience, Rehabilitation, Ophthalmology, Genetics and Maternal Child Health, University of Genova, Italy; 6 Ospedale Policlinico San Martino-IRCCS, Genoa, Italy; 7 Department of Experimental Medicine, Section of Human Physiology, University of Genoa, Italy; 8 Radboud University Medical Center, Department of Neurology, Donders Institute for Brain, Cognition and Behaviour, Nijmegen, The Netherlands; 9 Radboud University Medical Center, Department of Geriatric Medicine, Donders Institute for Brain, Cognition and Behaviour, Nijmegen, The Netherlands; 10 Department of Biomedical Sciences, Bioengineering Unit, University of Sassari, Sassari, Italy; 11 Interuniversity Centre of Bioengineering of the Human Neuromusculoskeletal System, Sassari, Italy; 12 Laboratory for Early Markers of Neurodegeneration, Center for the study of Movement, Cognition and Mobility, Neurological Institute, Tel Aviv Sourasky Medical Center, Israel; 13 Sackler Faculty of Medicine, and Sagol School of Neuroscience, Tel Aviv University, Israel; 14 Department of Physical Therapy, Sackler School of Medicine and Sagol School of Neuroscience, Tel Aviv University, Israel; 15 Rush Alzheimer’s Disease Center and Department of Orthopaedic Surgery, Rush University Medical Center, Chicago, Illinois; 16 Newcastle upon Tyne Hospitals NHS Foundation Trust, UK

**Keywords:** Falls, Parkinsons, Physical activity, Exercise, Wearable Technology

## Abstract

**Background:**

Physical activity is linked to many positive health outcomes, stimulating the development of exercise programs. However, many falls occur while walking and so promoting activity might paradoxically increase fall rates, causing injuries, and worse quality of life. The relationship between activity exposure and fall rates remains unclear. We investigated the relationship between walking activity (exposure to risk) and fall rates before and after an exercise program (V-TIME).

**Methods:**

One hundred and nine older fallers, 38 fallers with mild cognitive impairment (MCI), and 128 fallers with Parkinson’s disease (PD) were randomly assigned to one of two active interventions: treadmill training only or treadmill training combined with a virtual reality component. Participants were tested before and after the interventions. Free-living walking activity was characterized by volume, pattern, and variability of ambulatory bouts using an accelerometer positioned on the lower back for 1 week. To evaluate that relationship between fall risk and activity, a normalized index was determined expressing fall rates relative to activity exposure (FRA index), with higher scores indicating a higher risk of falls per steps taken.

**Results:**

At baseline, the FRA index was higher for fallers with PD compared to those with MCI and older fallers. Walking activity did not change after the intervention for the groups but the FRA index decreased significantly for all groups (*p* ≤ .035).

**Conclusions:**

This work showed that V-TIME interventions reduced falls risk without concurrent change in walking activity. We recommend using the FRA index in future fall prevention studies to better understand the nature of intervention programs.

Each year, over 30% of older adults (>65 years) fall at least once, a figure that increases to 40%–80% among people with mild cognitive impairment (MCI) or Parkinson’s disease (PD) (1,2). Falls are the primary cause of injurious death in older adults and lead to loss of functional independence and poor quality of life, including depression, social isolation, and reduction in walking activity, all this with a significant societal burden (3). Major research efforts have been directed to reduce falls risks, with varying levels of success (4).

Research into the circumstances of falls clearly shows that falls occur mostly while people are walking (being physically active) (5,6). Time spent upright and while walking therefore increases the exposure to the risk of a fall, highlighting the dynamic nature of fall risk (5–10). Maintaining walking activity, however, is important for a wide variety of reasons (eg, to maintain or improve cardiovascular fitness) and is, therefore, the focus of many clinical trials (11,12). A concern is that promoting physical activity might paradoxically be offset by an increase in fall rates, thus causing injuries and worse quality of life. Yet, the main outcome measure in falls trials is to document the occurrence and frequency of a fall, without taking into account any concurrent changes in walking-related exposure. For example, a marked reduction in falls after an intervention might theoretically be explained by a further loss of mobility, for example, because patients became more fearful of falling and hence more sedentary. Understanding the relationship between activity levels and falls rate is, therefore, fundamental for identifying clinically relevant reduction in falls risk.

To date, however, the understanding of this relationship is not clear. Studies reported conflicting results where older adults with higher levels of activity sustained fewer falls (13), as opposed to others where higher levels of activity were associated with an increase in falls (14). For people with PD, the situation is even more complicated with increasing disease severity. Specifically, people in early stages of PD initially have few falls per unit of activity, but show a concomitant increase over time as physical activity levels remain the same but postural stability and the safety of gait worsen with disease progression (11). The final stage is a reduction in falls due to progressive immobility as patients become confined to their wheelchair or bed (11). Examining falls risk and activity exposure (number of falls in relation to amount of activity) may offer a better understanding of falls risk, potentially showing a higher falls risk exposure for less active people both in older adults (8,13,15) and in people with PD (16). Previous work has looked at this aspect by using various metrics (eg, number of fallers per 1,000 physically active person-days (15), falls per 100 hours walked or falls per individual physical activity exposure time (13,16), falls over total number of steps taken (10)) in cross-sectional or in descriptive studies characterizing falls risk in various disease groups. Utility of these metrics in intervention studies has not been addressed. Our work extends this concept by proposing a normalized and customized index to quantify falls risk and activity exposure and applies it to an intervention study.

The main aims of this study were therefore twofold. First, to investigate the relationship between exposure to risk (activity) and falls events, by comparing three groups of older adults with falls (falls in the older adults; people with MCI; and people with PD). We measured activity objectively and continuously using wearable technology to provide a quantitative measure of walking activity in free-living conditions, allowing the risk/activity relationship to be more fully explicated (17–20). Second, to explore the relationship in the context of a clinical trial to better understand the impact of an exercise intervention aimed at reducing falls on this relationship. We used data from a randomized clinical trial (RCT), which was an intensive treadmill-based intervention with and without virtual reality (V-TIME). The results showed a significant reduction in number of falls in older adults from three groups (older fallers, people with PD and people with MCI) (21). In order to describe the relationship between exposure to falls risk (activity levels) and falls events per se, we propose an index that is customizable and normalized to walking activity to better explore the relationship of falls risk (rate of falls) relative to exposure (walking activity): the Falls Rate to Activity (FRA) index. More specifically, we hypothesized that the falls rate relative to activity exposure would be higher in people with PD compared to older adults and to people with MCI. We further hypothesized that the V-TIME intervention—which aimed to reduce falls—would decrease the FRA index in all three groups (ie, that the reduction in falls reported previously following this intervention (22) would not be explained solely by a concurrent reduction in total walking activity, but rather by an intrinsic effect on fall risks per se).

## Methods

### Participants

Older adult fallers (older fallers, people with MCI and people with PD) were enrolled in the V-TIME study at five clinical centers across five countries (22). Participants were included if they were aged 60−90 years, able to walk for at least 5 minutes unassisted, on stable medication for the past month, and self-reported two or more falls within 6 months before screening. In addition to these criteria, people with MCI were included only if they had a score of 0.5 on the Clinical Dementia Rating scale. A detailed list of inclusion and exclusion criteria is described elsewhere (22). All decisions about eligibility were made before randomization.

Demographic data were recorded for each participant. V-TIME study testing took place at the five clinical sites. The study was conducted according to the declaration of Helsinki and was approved by local Ethics Committees. All participants signed an informed consent form prior to testing (22).

### Randomization, Intervention Protocol, and Falls Data Capture

Participants were randomly assigned to a 6-week, three times per week, 40-minute treadmill training only (TT) or treadmill training plus virtual reality (TT + VR) interventions. Study design and interventions are described in detail elsewhere (21,22).

Falls rate was recorded at baseline and during the 6 months after the end of the training. A fall was defined as “an unexpected event in which the participant comes to rest on the ground, floor or lower level” (23). According to their preference, participants were provided with a falls calendar as a paper version, web-based calendar, or a smartphone application. Information logged in the online or smartphone-based calendar was automatically uploaded to a database, whereas the paper calendars were posted back to the sites at which participants were recruited each month via preaddressed envelopes. Research staff contacted all participants every month to maximize compliance (21,22).

### Free-Living Walking Activity Data Collection: Protocol

Participants were tested four times: preintervention (T1), and postintervention (after 1 week [T2], 1 month [T3], and 6 months [T4]). At the end of each time point, participants were asked to wear a tri-axial accelerometer-based wearable device (Axivity AX3, York, UK; dimensions: 23.0 × 32.5 × 7.6 mm; weight: 11 g; accuracy of the quartz-stabilized real-time clock: 20 parts per million) for 1 week; this device has been validated for suitability in capturing high-resolution data akin to human movement (24,25). The wearable was located on the fifth lumbar vertebra with a hydrogel adhesive (PAL Technologies, Glasgow, UK) and covered with the Hypafix bandage for extra support. The wearable was programmed to capture data for 7 days at 100 Hz and at an acceleration range of ±8 g. Participants were asked to continue their daily activities as usual and not to change their routine. Upon completion of recording, the participants removed the device and posted it back to the researcher (18).

### Data Processing and Analysis

#### Data processing and variable extraction—free-living data

Once the wearable was received, data were downloaded and segmented (per calendar day). For each day, individual ambulatory bouts (ABs) were extracted via MATLAB, where a “bout” was defined as the continuous length of time spent walking (19). AB were detected by applying selective thresholds on the standard deviation and the magnitude vector of the triaxial accelerations (26). All ABs greater than 10 seconds (minimum bout length) were taken into account for the analysis (17–19,27–31); a threshold of 2.5 seconds was set for the maximum resting period between consecutive ABs; in other words, if there was a break of 2.5 seconds between two walking episodes, for example, it was considered as one AB (26).

Custom-made MATLAB programs were used to extract outcome measures. Pooled 7-day data were used for quantifying outcomes. Outcome measures were described according to a broad framework that captured the overall volume (amount), pattern, and variability of walking activity (32). The outcomes specifically were the following: volume of walking (eg, number of steps per day), mean AB length was generated based on the AB detected over the 7 days. In addition, a set of nonlinear descriptors were also derived: (i) the pattern of ABs was quantified using alpha (α), which describes ABs distribution, evaluating the ratio of short to long ABs (eg, a high alpha means that the total walking time is made up of proportionally short ABs compared to long ABs) and (ii) the within AB variability (*S*_*2*_) of bout length estimated using a maximum likelihood technique (33,34).

### An Index of Falls Rate to Activity (FRA index)

We quantified the falls risk taking into account changes in number of falls and walking activity (exposure) pre- and postintervention. We defined this as the “Falls Rate to Activity Index (FRA index),” a measure of exposure-adjusted incidence of falls. The FRA index can be applied flexibly because the denominator can be changed to suit the specific protocol of any falls study. For this study, we normalized falls over a set measure of walking exposure (100,000 steps) to enable comparability between studies. The FRA index describes the ratio between the number of falls reported in a given period of time (*N* days) prior to a walking activity assessment, and outcomes of the volume of walking activity (total number of steps per day, equation 1), normalized per 100,000 steps (10,13,15,16,35).

In the case of V-TIME study, the FRA index describes the ratio between the number of falls reported in the previous 180 days (6 months, *N* = 180) of the free-living walking assessment, and outcomes of the volume of walking activity (total number of steps per day, equation 2) normalized per 100,000 steps.

$$\begin{array}{l} FRA\;index=\frac{Number\;of\;Falls\;(past\;N\;days)}{N\;days\;*\;Total\;number\;of\;steps\;per\;day}\;*\;100,000 \end{array}$$ (1)

$$\begin{array}{l} FRA\;index=\frac{Number\;of\;Falls\;(past\;180\;days)}{180\;\;\;days\;*Total\;number\;of\;steps\;per\;day}*\;100,000 \end{array}$$ (2)

To aid the interpretation of this index, it is directly proportional to number of falls and indirectly proportional to activity (number of steps taken per day), so a reduction in falls (or an increase in activity) would reflect a numerical decrease in the index, thus reflecting a decrease in falls risk per 100,000 steps taken. On the other hand, a rise in falls (or a concurrent decrease in activity) would translate into a numerical increase of the index, thus reflecting a worsening falls risk per 100,000 steps taken. The index adjusts for activity levels, taking into account that a decrease in numbers of falls and a similar decrease in activity would not show a decrease in the index (so in falls risk) which would remain at the same level. So for example, if a person halved number of falls but at the same time halved his activity, the index and therefore falls risk would remain the same. We quantified this index separately, for each time point (preintervention [T1] and 6 months postintervention [T4]), using walking activity and falls rate data from T1 (preintervention/baseline assessment) and T4 (6 months after the intervention assessment) in order to consider the same period of time (6 months) for determining fall risk, with no overlap.

The advantage of the FRA index is that using a daily measure of volume of walking (ie, steps per day), it gives flexibility in relation to the period of time falls have been recorded/reported (eg, 6 months in our case) and with the additional normalization over 100,000 steps, it facilitates comparability across studies.

### Statistical Analysis

Statistical analysis was carried out using SPSS v19 (IBM). Normality of data and homoscedasticity were tested with Shapiro-Wilk test and Levene’s Test of Equality of Variances respectively. Descriptive statistics were reported as means and standard deviations (*SD*).

We compared changes in activity levels. The effect of group (“pooled data”), time and type of intervention were examined using random effects linear mixed-models (RELM). We included type of intervention (TT vs TT + VR) as a fixed-effect and time (T1 vs T4), group (older fallers vs people with PD vs people with MCI), age and sex as covariates; random intercepts were modeled (36). We tested pre-postintervention change in the FRA index: we used nonparametric repeated measure tests as the index was not normally distributed. Analysis of outcome measures and FRA index was performed on ambulatory bouts longer than 10 seconds.

Following the methodology of the main V-TIME study, we include site as a covariate; the effect was not significant and site was therefore not included in any of the final models (data not shown). We referred to the modified intention-to-treat population used for the efficacy analyses as the full analysis set, which adhered as closely to the intention-to-treat principle as was possible. The full analysis set included all participants who underwent randomization, satisfied eligibility criteria, had at least three training sessions, and had any assessments during the 6-month follow-up period. According to the intention-to-treat principles, any participants who were randomly assigned to a group but discontinued the study before 6 months of follow-up were included into the full analysis set. Missing data resulting from dropouts, technical problems, and human errors were not imputed as per main study (22). We used a threshold of *p* < .05 to guide statistical interpretation for the main effects, while a Bonferroni corrected threshold (*p* < .0167) was used to account for the multiple comparisons (three groups).

## Results

Of the 282 participants who completed training and were included in the main study full analysis set (22), 275 completed the free-living walking assessment (133 in the TT group and 142 in the TT + VR group (22), Supplementary Figure 1). The distribution of the three participant subgroups (109 older fallers, 38 participants with MCI and 128 participants with PD) was similar between the two intervention groups (52 in TT group vs 57 in the TT + VR group for older fallers; 19 vs 19 for participants with MCI; 62 vs 66 for participants with PD). Participants’ baseline clinical and demographic characteristics are presented in Table 1. People with PD fallers were younger than older fallers and people with MCI and included proportionally fewer women (37%) than older fallers (78%) and people with MCI (68%). Baseline statistical differences between the randomized groups are not reported (37).

**Table 1.** Clinical and Demographic Characteristics for Idiopathic Older Fallers, Fallers with MCI and Fallers with PD at Baseline

Characteristics	Older Fallers (*n* = 109) Mean (*SD*)	Fallers with MCI (*n* = 38) Mean (*SD*)	Fallers with PD (*n* = 128) Mean (*SD*)
Intervention (TT/TT + VR)	52/57	19/19	62/66
Female (n, %)	85, 78%	26, 68%	47, 37%
Age (years)	75.93 (6.22)	78.03 (6.21)	71.68 (6.43)
BMI (kg/m^2^)	26.02 (4.26)	26.28 (4.56)	25.96 (3.62)
MMSE	28.52 (1.36)	25.95 (2.4)	28.07 (1.68)
Hoehn and Yahr (HY) stage (%)	-	-	HY 2–48% HY 2.5–10% HY 3–42%
MDS-UPDRS III	-	-	30.37 (16.96)
Freezing of gait (%, Score)	-	-	59%, 9.16 (9.33)
FES-I (16–64)	28.76 (7.67)	30.38 (10.11)	34.98 (11.73)

*Note*: BMI = Body Mass Index; FES-I = Falls Efficacy Scale; MCI = Mild Cognitive Impairment; MMSE = Mini-Mental State Examination; MDS-UPDRS III = Movement Disorder Society Unified Parkinson’s Disease Rating Scale part III; PD = Parkinson’s disease.

### Differences Between Groups Prior to Intervention

#### Walking activity

First, we describe the activity levels for each cohort. There was a significant effect of group (EF vs MCI vs PD), where the volume of walking (total walking time per day, percentage [%] of walking time per day, number of bouts, and steps per day) was lower in people with MCI and people with PD compared to older fallers (Table 2, *p* ≤ .012).

**Table 2.** Free-Living Outcomes for Participants Grouped as OF, Fallers with MCI and Fallers with PD, Preintervention (T1) and 6 Months Postintervention (T4)

ABs > 10 s	T1 (preintervention)			T4 (6 months postintervention)		
TT	Older Fallers	Fallers with MCI	Fallers with PD	Older Fallers	Fallers with MCI	Fallers with PD
**Volume**						
Total Walking Time per Day (Min)^a^	128.6 (58.3)	125.2 (48.7)	117.3 (59.1)	119.7 (64.0)	116.4 (61.8)	116.2 (65.1)
Percentage of Walking Time^a^	8.9 (4.1)	8.7 (3.4)	8.2 (4.1)	8.3 (4.5)	8.1 (4.3)	8.1 (4.5)
Number of steps per Day^a^	9,833 (4,793)	9,354 (3,712)	8,874 (4,538)	8,993 (5,092)	8,765 (4,723)	8,654 (4,638)
Bouts per Day^a^	264 (105)	268 (98)	233 (106)	250 (94)	254 (129)	229 (116)
Mean Bout Length (sec)^b^	28.9 (5.7)	27.8 (4.4)	29.8 (5.8)	28.0 (6.4)	27.2 (3.7)	30.4 (6.3)
**Variability**						
Variability (S_2_)	0.620 (0.095)	0.605 (0.082)	0.630 (0.094)	0.599 (0.094)	0.595 (0.076)	0.636 (0.100)
**Pattern**						
Alpha (α)	2.621 (0.27)	2.650 (0.23)	2.553 (0.299)	2.658 (0.258)	2.619 (0.144)	2.515 (0.247)
**TT + VR**	Older Fallers	Fallers with MCI	Fallers with PD	Older Fallers	Fallers with MCI	Fallers with PD
**Volume**						
Total Walking Time per Day (Min)^a^	142.2 (59.9)	113.4 (41.2)	117.8 (62.3)	137.7 (64.8)	107.4 (36.7)	127.9 (84.3)
Percentage of Walking Time^a^	9.9 (4.2)	7.9 (2.9)	8.2 (4.3)	9.6 (4.5)	7.5 (2.6)	8.9 (5.9)
Number of steps per Day^a^	10,802 (4,700)	8,252 (3,218)	9,000 (4,535)	10,423 (5,274)	8,032 (2,942)	9,468 (5,576)
Bouts per Day^a^	288 (97)	261 (84)	242 (110)	282 (99)	248 (86)	254 (107)
Mean Bout Length (seconds)^b^	29.2 (6.0)	25.9 (3.1)	28.4 (4.9)	28.3 (6.7)	26.0 (2.5)	29.0 (7.0)
**Variability**						
Variability (S_2_)	0.612 (0.091)	0.562 (0.063)	0.606 (0.083)	0.594 (0.108)	0.569 (0.050)	0.607 (0.104)
**Pattern**						
Alpha (α)	2.555 (0.209)	2.690 (0.237)	2.587 (0.274)	2.597 (0.276)	2.691 (0.185)	2.557 (0.271)

*Note*: Longitudinal data are presented for each intervention arm, for all ambulatory bouts longer than 10 seconds (ABs > 10 s). Values are presented as mean (*SD*).

OF = Older Fallers; MCI = Mild cognitive impairment; PD = Parkinson’s disease; TT = Treadmill training only intervention; TT + VR = Treadmill training plus virtual reality intervention.

^a^Significant (*p* < .05) Group effect (OF vs MCI vs PD); ^b^significant (*p* < .05) Group × Intervention interaction.

### Falls Rate and Activity Exposure Index (the FRA index)

People with PD had a higher number of falls compared to both older fallers and people with MCI (*p* ≤ .036, Table 3). People with PD had a significantly higher FRA index compared to older fallers and people with MCI (significant effect of group by FRA index; *p* = .043, Table 3). Preintervention, people with PD experienced on average 2 falls for every 100,000 steps versus 0.6 falls for older fallers and 0.2 falls for people with MCI (Table 3).

**Table 3.** FRA Index Together with Number of Falls and Step Per Day Results Preintervention (T1) and 6 Months Postintervention (T4) for Idiopathic OF, Older Fallers with MCI, and Fallers with PD

ABs > 10 s	T1 (preintervention)			T4 (6 months postintervention)		
TT	Older Fallers	Fallers with MCI	Fallers with PD	Older Fallers	Fallers with MCI	Fallers with PD
FRA Index^a^						
Mean (*SD*)	0.640 (2.839)^b^	0.210 (0.181)^b^	2.440 (5.941)^b^	0.074 (0.191)^b^	0.185 (0.5)^b^	2.177 (7.846)^b^
Median (IQR)	0.146 (0.209)	0.159 (0.159)	0.271 (0.972)	0 (0.054)	0 (0.089)	0.061 (0.68)
Number of Falls^a^						
Mean (*SD*)	3.278 (2.476)	2.857 (1.352)	26.478 (67.962)	0.815 (1.854)	1.286 (2.61)	14.955 (57.392)
Median (IQR)	2 (1)	2 (1.5)	3 (5)	0 (1)	0 (2)	1 (6)
Number of steps per Day^a^	9,833 (4,793)	9,354 (3,712)	8,874 (4,538)	8,993 (5,092)	8,765 (4,723)	8,654 (4,638)
**TT + VR**						
FRA Index^a^						
Mean (*SD*)	0.487 (1.717)^b^	0.248 (0.157)^b^	1.573 (4.732)^b^	0.257 (1.35)^b^	0.098 (0.209)^b^	1.052 (4.472)^b^
Median (IQR)	0.127 (0.193)	0.214 (0.145)	0.261 (0.292)	0 (0.105)	0 (0.077)	0.104 (0.378)
Number of Falls^a^						
Mean (*SD*)	8.017 (28.493)	3.2 (1.576)	17.691 (51.06)	4.75 (27.658)	2.4 (4.86)	6.971 (17.225)
Median (IQR)	2 (1)	2.5 (2.75)	3 (3)	0.5 (2)	1 (1.75)	2 (5.75)
Number of steps per Day^a^	10,802 (4,700)	8,252 (3,218)	9,000 (4,535)	10,423 (5,274)	8,032 (2,942)	9,468 (5,576)

*Note*: Longitudinal data are presented for each intervention arm for all ambulatory bouts longer than 10s (ABs > 10 s).

OF = Older Fallers; FRA = Falls Rate to Activity; MCI = Mild Cognitive Impairment; IQR = Interquartile range; PD = Parkinson’s disease; SD = Standard deviation; TT = Treadmill training only intervention; TT + VR = Treadmill training plus virtual reality intervention.

^a^Significant (*p* < .05) Group effect (OF vs MCI vs PD); ^b^significant (*p* < .05) Time effect.

### Changes in Response to Intervention (Longitudinal data)

#### Walking activity

Walking activity did not change over time (main effect for Time, *p* > .068) irrespective of intervention type. In addition, there was no main intervention effect, significant Group × Time or Intervention × Time interactions.

### Falls Rate and Activity Exposure Index (the FRA index)

**Figure 1.**
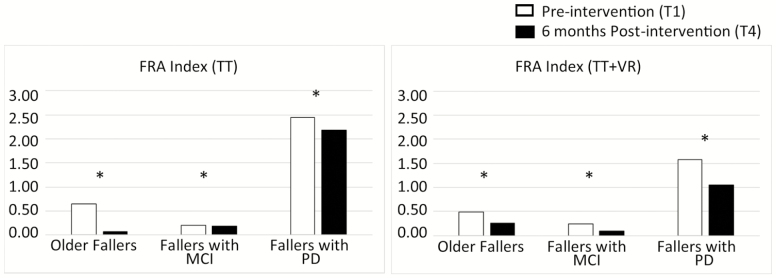
Falls Rate to Activity Index (FRA index) results preintervention (T1, white bars) and 6 months postintervention (T4, black bars) for idiopathic Older Fallers, older fallers with Mild Cognitive Impairment (MCI), and fallers with Parkinson’s disease (PD), evaluated in free-living conditions. * represents post-hoc significant differences (*p* values < 0.05). TT = Treadmill training only intervention; TT + VR = Treadmill training plus virtual reality intervention.

The FRA index significantly decreased for all groups following both interventions, showing that falls risk reduced without this being explained by a concurrent reduction in walking activity levels (Table 3, Figure 1, *p* ≤ .035). For the whole group of participants (older fallers + people with PD + people with MCI), the FRA index significantly reduced from 0.9 to 0.6 (*p* < .001). Overall, the TT + VR intervention reduced the FRA index by 39% and the TT intervention by 26%. For all three groups, irrespective of intervention, the FRA index significantly decreased postintervention (*p* ≤ .002, older fallers from 0.6 to 1.7, people with MCI from 0.2 to 0.1 and people with PD from 2.0 to 1.6, Table 3). To quantify the reduction of this falls risk, we will provide one example here. Preintervention (T1), older fallers on average experienced 0.6 falls for every 100,000 steps, and this decreased to 0.2 falls after the intervention (T4), thereby significantly reducing this adjusted falls risk by approximately two thirds (Table 3). Results were similar also when we used T2, T3, and averaged (T2, T3, and T4) total number of steps per day values for the denominator of the index (Supplementary Table 1).

## Discussion

Our primary aim was to evaluate the relationship between exposure to falls risk (total activity levels) and falls events (expressed as the normalized FRA index) in three groups of older adults with falls, and to explore the effect of an intervention aimed to reduce falls (V-TIME) using this FRA index as outcome. The FRA index improved postintervention for all groups. This was driven by a true reduction in falls frequency, as activity levels remained the same. These findings are important as they provide a more nuanced approach to measuring falls status than usual measures. As such, the FRA index paints a clearer picture of the true impact of interventions aimed at reducing falls risk.

### Differences in Falls Risk Relative to Activity Levels Across Different Cohorts of Fallers

Walking activity was lower in people with PD and with MCI compared to older fallers, while falls rates were higher in people with PD. In line with previous research, we also found that people with PD and with MCI walked less than older fallers (18,38). Importantly, we found that people with PD had the highest FRA index relative to older fallers and people with MCI, confirming the higher falls risk in people with PD in the older adults’ spectrum, but now even when time spent being active is considered (1,21). Specifically, preintervention people with PD experienced two falls every ~100,000 steps compared to 0.6 for older fallers and 0.2 for people with MCI. Although it is difficult to compare our study with others due to differences in the quantification of falls incidence and activity levels, this finding is in agreement with previous studies looking at fall incidents and exposure to activity in people with PD (16).

Previous studies in older adults and people with PD showed that including metrics of exposure to activity (falls per 100 hours walked) and falls incidence might be a sensitive measure of falls risk (13,16). A key difference in our work is that the FRA index quantifies activity exposure using quantified numbers of steps per day, rather than an estimate of hours spent walking (13,15), this aids the clinical interpretability of the results as often guidelines on recommended walking activity levels are expressed as the number of steps (per day). But another benefit of the outcome measure “falls per activity” captured by the FRA index is that it is not limited to a specific denominator (steps or activity duration); depending on the purpose of the analysis and application, the denominator has the capability of flexibility in order to target specific activity metrics which may change with guidelines and field of application (eg, some activity guidelines express the recommendation as durations).

We consider the FRA index a preliminary but important step toward addressing the past and ongoing work on fall risk models and the recognized need to consider exposure (activity) and context (environment) in addition to intrinsic factors (eg, age, sex, etc.) to fully characterize falls risk (8,39).

### The Effect of an Exercise-Based Intervention (V-TIME) on Falls Risk

To understand the effect of exercise, it was necessary to first determine changes in activity levels following the intervention. We showed that walking activity (for any of its measures) did not change significantly following the intervention, irrespective of group or intervention arm. We further showed that the FRA index decreased significantly for all groups, irrespective of the intervention, showing a postintervention decrease in falls, driven by a drop in falls rate rather than a decrease in walking activity (exposure). In contrast with what was shown in the main study, we did not find an intervention effect for the FRA index (TR vs TT + VR). This may be due to the reduced number of participants that were included in this analysis, limited by the availability of free-living walking data. Participants managed to reduce their falls risk by decreasing the number of falls without reducing their walking activity/exposure. The decrease in the FRA index, therefore, indicated the reduction in the number of falls, taking into account exposure to activity. The V-TIME trial was therefore successful in reducing falls and also significantly reducing falls risk.

### Changing Walking Activity

It could be argued that the optimal result of an exercise-based intervention would be a reduction in falls accompanied by an increase in activity. Note that the V-TIME intervention was focused exclusively on a lab-based exercise intervention aimed to reduce falls, but no attempt was made to increase physical activities in the patients’ own home environment in between the hospital visits. In this regard, an important finding is that the volume of walking activity did not get lower in any of the three groups, but was actually maintained; this can be seen as a positive outcome because previous studies showed the importance of maintaining activity levels to decrease falls risk (11,12). It is also important to note that over a regular 6-month observation period, that is, without any intermittent intervention such as V-TIME, one might expect to see spontaneous reductions in walking activity in PD patients (40) and older adults.

### Limitations

Further work is needed to assess the merits of our initial analysis, especially in studies targeting increases in walking behavior. In addition, the examination of other diseases with a history of falls will allow us to identify specific interventions/rehabilitation strategies for reducing falls risk while maintaining walking activity levels. Future work also needs to address specific technical limitations of instrumented free-living walking assessment (eg, examining the effect of merging short ambulatory bouts on results) for considering a broader impact of the interventions on walking behavior. We recorded walking activity for only 1 week, which may not be enough to capture a reliable impression of a person’s true physical activity levels over longer periods of time (eg, subjects may have behaved differently during the week of observation). Inclusion of walkability data of towns should also be considered in future studies, in fact walkability has recently been shown to be an important factor impacting walking activity and falls risk (41,42). Finally, the preintervention falls rate was based on participants’ recall, while postintervention falls rate on falls diary, this may have impacted on the reported preintervention outcomes as asking people to recall falls might lead to underreporting (43). Ideally, the pairing of objective walking activity and objective falls detection methods would improve sensitivity of this type of outcome (eg, FRA index).

## Conclusions

Our findings suggest the utility and sensitivity of using a customizable and normalized FRA index to better understand and disentangle the effect of clinical trials testing the efficacy of interventions on fall rates and walking activity levels in populations at risk of falling. In our specific example, the V-TIME intervention successfully reduced falls risks while maintaining walking activity levels across different cohorts of older adult fallers.

## Supplementary Material

glaa007_suppl_Supplementary_Figure_1

glaa007_suppl_Supplementary_material
