# Supplementary figures and images for: Falls Risk in Relation to Activity Exposure in High-Risk Older Adults
Source: J Gerontol A Biol Sci Med Sci. 2020 Jan 16;75(6):1198–205. doi: 10.1093/gerona/glaa007 (PMC7243591; doi:10.1093/gerona/glaa007)

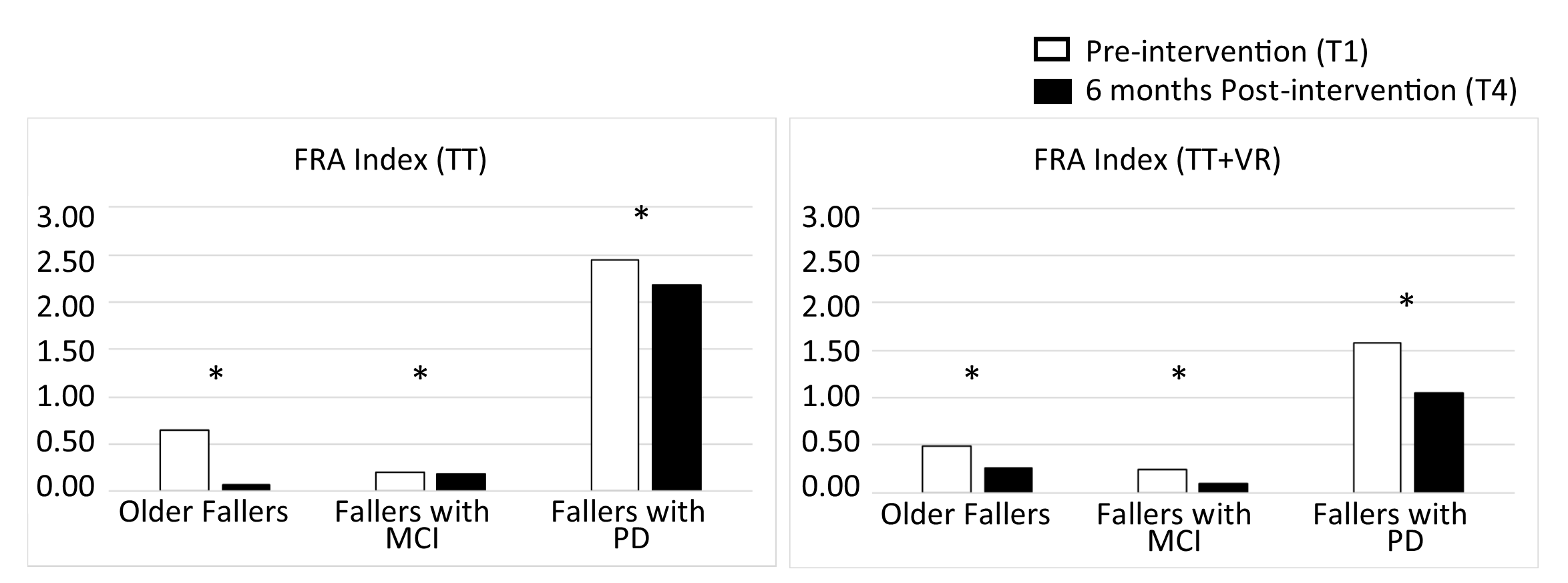

Supplement: glaa007_suppl_Supplementary_Figure_1 [file glaa007_suppl_supplementary_figure_1.png]
