# Supplementary material for: Falls Risk in Relation to Activity Exposure in High-Risk Older Adults
Source: J Gerontol A Biol Sci Med Sci. 2020 Jan 16;75(6):1198–205. doi: 10.1093/gerona/glaa007 (PMC7243591; doi:10.1093/gerona/glaa007)
Supplement: glaa007_suppl_Supplementary_material [file glaa007_suppl_supplementary_material.docx]

**Supplementary Table 1:**

Falls Rate to Activity Index (FRA Index) results pre-intervention (T1) and post-intervention (1week post-intervention (T2), 1 month post-intervention (T3) and averaged (T2, T3 and T4 (6 months post-intervention)) total number of step per day values) for idiopathic Older Fallers, fallers with Mild Cognitive Impairment (MCI) and fallers with Parkinson’s disease (PD). Data are presented for each intervention arm for all ambulatory bouts longer than 10s (ABs > 10s).

| **ABs>10s** | T1 (pre-intervention) | | | T2 (1 week post-intervention) | | | |
| --- | --- | --- | --- | --- | --- | --- | --- |
| **TT** | Older Fallers ^b^ | Fallers with MCI ^b^ | Fallers with  PD ^b^ | Older Fallers ^b^ | Fallers with MCI ^b^ | Fallers with  PD ^b^ |  |
| FRA Index ^a^ |  |  |  |  |  |  |  |
| Mean (SD) | 0.640 (2.839) | 0.210 (0.181) | 2.440 (5.941) | 0.084 (0.251) | 0.137 (0.402) | 1.129 (4.163) |  |
| Median (IQR) | 0.146 (0.209) | 0.159 (0.159) | 0.271 (0.972) | 0 (0.047) | 0 (0.093) | 0.102 (0.443) |  |
| **TT+VR** |  |  |  |  |  |  |  |
| FRA Index ^a^ |  |  |  |  |  |  |  |
| Mean (SD) | 0.487 (1.717) | 0.248 (0.157) | 1.573 (4.732) | 0.376 (1.607) | 0.192 (0.415) | 0.482 (1.064) |  |
| Median (IQR) | 0.127 (0.193) | 0.214 (0.145) | 0.261 (0.292) | 0 (0.122) | 0.041 (0.119) | 0.124 (0.448) |  |
|  | T1 (pre-intervention) | | | T3 (1 moth post-intervention) | | | |
| **TT** | Older Fallers ^b^ | Fallers with MCI ^b^ | Fallers with  PD ^b^ | Older Fallers ^b^ | Fallers with MCI ^b^ | Fallers with  PD ^b^ |  |
| FRA Index ^a^ |  |  |  |  |  |  |  |
| Mean (SD) | 0.640 (2.839) | 0.210 (0.181) | 2.440 (5.941) | 0.048 (0.107) | 0.124 (0.342) | 1.615 (4.953) |  |
| Median (IQR) | 0.146 (0.209) | 0.159 (0.159) | 0.271 (0.972) | 0 (0.05) | 0 (0.094) | 0.078 (0.318) |  |
| **TT+VR** |  |  |  |  |  |  |  |
| FRA Index ^a^ |  |  |  |  |  |  |  |
| Mean (SD) | 0.487 (1.717) | 0.248 (0.157) | 1.573 (4.732) | 0.263 (1.291) | 0.166 (0.322) | 0.48 (1.028) |  |
| Median (IQR) | 0.127 (0.193) | 0.214 (0.145) | 0.261 (0.292) | 0 (0.122) | 0.045 (0.131) | 0.12 (0.437) |  |
|  | T1 (pre-intervention) | | | Averaged (T2-T3-T4) | | | |
| **TT** | Older Fallers ^b^ | Fallers with MCI ^b^ | Fallers with  PD ^b^ | Older Fallers ^b^ | Fallers with MCI ^b^ | Fallers with  PD ^b^ |  |
| FRA Index ^a^ |  |  |  |  |  |  |  |
| Mean (SD) | 0.640 (2.839) | 0.210 (0.181) | 2.440 (5.941) | 0.059 (0.138) | 0.139 (0.397) | 1.301 (4.608) |  |
| Median (IQR) | 0.146 (0.209) | 0.159 (0.159) | 0.271 (0.972) | 0 (0.054) | 0 (0.093) | 0.081 (0.448) |  |
| **TT+VR** |  |  |  |  |  |  |  |
| FRA Index ^a^ |  |  |  |  |  |  |  |
| Mean (SD) | 0.487 (1.717) | 0.248 (0.157) | 1.573 (4.732) | 0.257 (1.251) | 0.175 (0.36) | 0.483 (1.003) |  |
| Median (IQR) | 0.127 (0.193) | 0.214 (0.145) | 0.261 (0.292) | 0 (0.116) | 0.047 (0.129) | 0.117 (0.522) |  |

TT: Treadmill training only intervention, TT+VR: Treadmill training plus virtual reality intervention, SD: Standard Deviation, IQR: Interquartile Range. OF: Older Fallers.

^a^ Significant (p<0.05) Group effect (OF vs MCI vs PD); ^b^ significant (p<.05) Time effect.

**Supplementary Figure 1:**

Free-living assessment trial profile. TT: Treadmill training only intervention, TT+VR: Treadmill training plus virtual reality intervention.

**
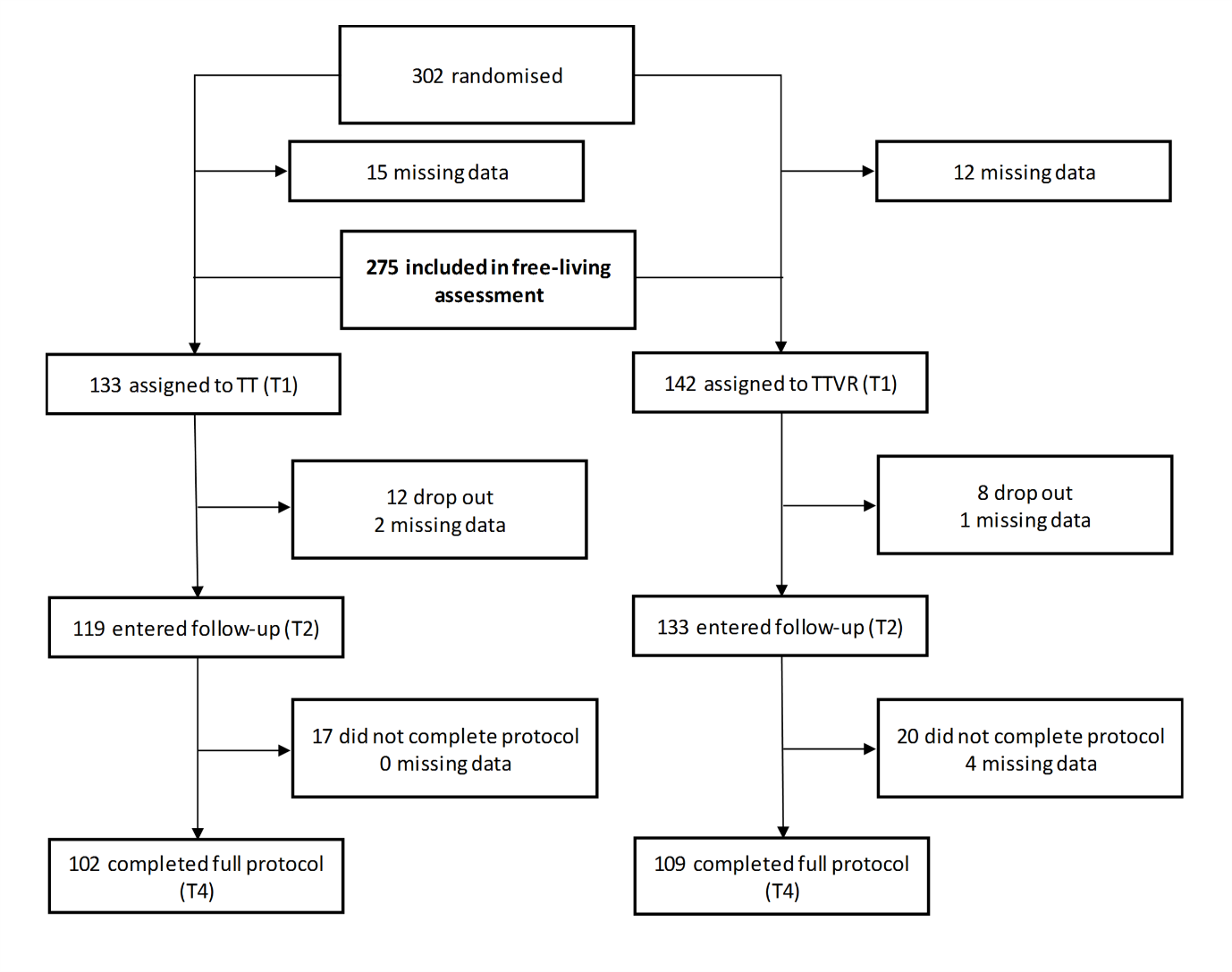
**
